# Rules for the Administration of Chloroform in Labor

**Published:** 1856-01

**Authors:** 


**RULES FOR THE ADMINISTRATION OF CHLOROFORM IN LABOR.**

1. Let the chloroform be pure. If rubbed on the hands, the smell should be fragrant, not pungent, like sulphuric ether. If inspired from the inhaler, there is a sense of warmth in the mouth, a fruity flavor, no pungency; if the strength of the vapor be sufficient, it will excite a slight cough; but if impure, the cough is irritating. Let the sponge of the inhaler be placed in warm water, and then rung perfectly dry. About thirty minims may be poured upon it, which is sufficient in the first instance.

2. When labor has commenced, do not interfere so long as the patient bears her pains well; if she be not teased with short, very severe, and inefficient pains, chloroform need not be given. If, on the contrary, the severity of the first stage be such, the anguish of the patient so great, that pain is evidently a cause of protraction, chloroform may be given with great benefit.

3. Always commence with a small dose, about thirty minims; if it agree with the patient no inconvenience is caused, but she will complain that it is doing no good; the quantity may then be increased, until on inhalation the exhibitor finds that she cannot take a full inspiration without cough.

4. In the second stage of labor, chloroform may be given when the head is approaching the perineum, or before then if the pains are intolerable. This may be known not merely by their greater intensity while the uterus is in action, but also by the restlessness of the patient in the intervals. She is watchful, dispirited, still crying, but in a more subdued tone, from pain and a feeling of soreness.

5. When the head arrives at the perineum, chloroform may be given in a fuller dose, if it have not already accumulated. The perineum yields more easily under its influence, and the severity of the pains is controlled without any loss of force. This rule applies especially to cases in which powerful forcing pains are acting against the perineum at the hazard of its laceration.

6. When operations are necessary, if they are not severe—as, for instance, some forceps operations—chloroform may be given in the same manner as in natural labor, but always after the instrument is applied.

If severe, it may be given as in surgicical operations, but not to the same extent. Hence an assistant is necessary who is conversant with the properties of this anæsthetic. It is obvious that the same person cannot operate and give simultaneously the full soporific dose of this agent.

7. The inhaler should be applied to the mouth just before the pain commences, two or three full inspirations taken, and the moment the action of the uterus ceases it should be withdawn. The inhaler should never be applied in the interval between the pains, and if used in the middle of a pain the cries of the patient blow away the vapor, and no relief is given.

8. When inhalation has been continued in this interrupted manner for some time, if any alteration be observed in the countenance or manner of the patient—if the face is flushed, or bloated, or tinged with a slight lividity—if she ramble or become hysterical, let the inhaler be removed, and the face of the patient fanned. Wait until the pains return to their original severity before renewing the inhalation, when it is probable that these symptoms will not return.

9. In some instances, the patient is very intolerant of her pain, and if given chloroform to relieve them, she becomes hysterical, crying, perhaps louder than before it was inhaled. In these instances it is better to induce sopor, which may easily be done, without stertor. For this purpose, a sponge and folded handkerchief applied to the nostrils is preferable to the inhaler. Whenever sopor is brought on, the closest attention should be given to the countenance—observe the irritability of the eyelids; to the respiration—notice its frequency, and especially stertor; to the pulse—mark its strength. The handkerchief should always be held at a distance at first, and be gradually brought nearer, but the sponge should never be applied quite close to the nostrils.

10. There should be the freest circulation of air in the apartment; and if, after delivery, there should be any feeling of faintness, ammonia in effervescence will relieve it.—p. 69.—*Nelson's Lancet.*

